# Supplementary material for: Functional Dynamics of Hexameric Helicase Probed by Hydrogen Exchange and Simulation
Source: Biophys J. 2014 Aug 19;107(4):983–90. doi: 10.1016/j.bpj.2014.06.039 (PMC4142241; doi:10.1016/j.bpj.2014.06.039)
Supplement: Document S1. Supporting Methods, Figs. S1–S6, and Tables S1 and S2 [file mmc1.pdf]

Supplementary information for "Functional dynamics of hexameric helicase probed by hydrogen exchange and simulation" by Radou, Dreyer, Tuma and Paci.

Verification of the assignment of the m/z peaks

To verify whether mismatches between experimental and predicted exchange kinetics of some fragments were due to misassignment, we double-checked the assignment of the raw MS peaks. The mass of all possible fragments of the primary sequence between length 5 and 40 aminoacids was calculated by summing the residue masses and adding the mass of a water molecule (18.01056 u), corresponding to the adding of OH and H during the hydrolysis of the new C- and N-terminus, respectively. Each mass experimentally detected was compared with the calculated ones. A threshold precision of 0.05u was used, i.e. the theoretical peak was considered to potentially match with the experimental one if $|m_{exp}-m_{th}|=\Delta m$ was lower than 0.05u (see Table S2).

Identification of the fragments located at the interface:

The variation of solvent accessible surface area from the monomeric to the hexameric form for a fragment *j*, ΔS_j, was quantified as:

$$\Delta S_j = \frac{1}{n_j} \sum_{i=m_j}^{m_j+n_j-1} \frac{S_i^{mono} - S_i^{hexa}}{S_i^{mono}} \qquad \text{S1}$$

where S_i^{mono} and S_i^{hexa} are the total solvent accessible surface area of the residue *i* in the monomeric and hexameric structure, respectively. Solvent accessible solvent accessible surface was calculated using NACCESS with a probe radius of 1.4 Å.

Quantification of the matching between the predicted and measured fragments kinetics

The overall deviation between the experimental and the simulated deuteration kinetics was estimated through the mean square deviation defined as

$$\chi^2 = \frac{1}{N}\sum_{j=1}^{N}\frac{1}{T_j}\sum_{t=1}^{T_j}(D_j^{exp}(t) - D_j^{sim}(t))^2 \qquad \text{S2}$$

where N is the total number of fragments, T_j the total number of experimental time points for the fragment *j*, D_j^{exp} the experimental deuterium fraction of the fragment *j* at time *t* and D_j^{sim} the simulated deuterium fraction of the fragment *j* at time *t*.

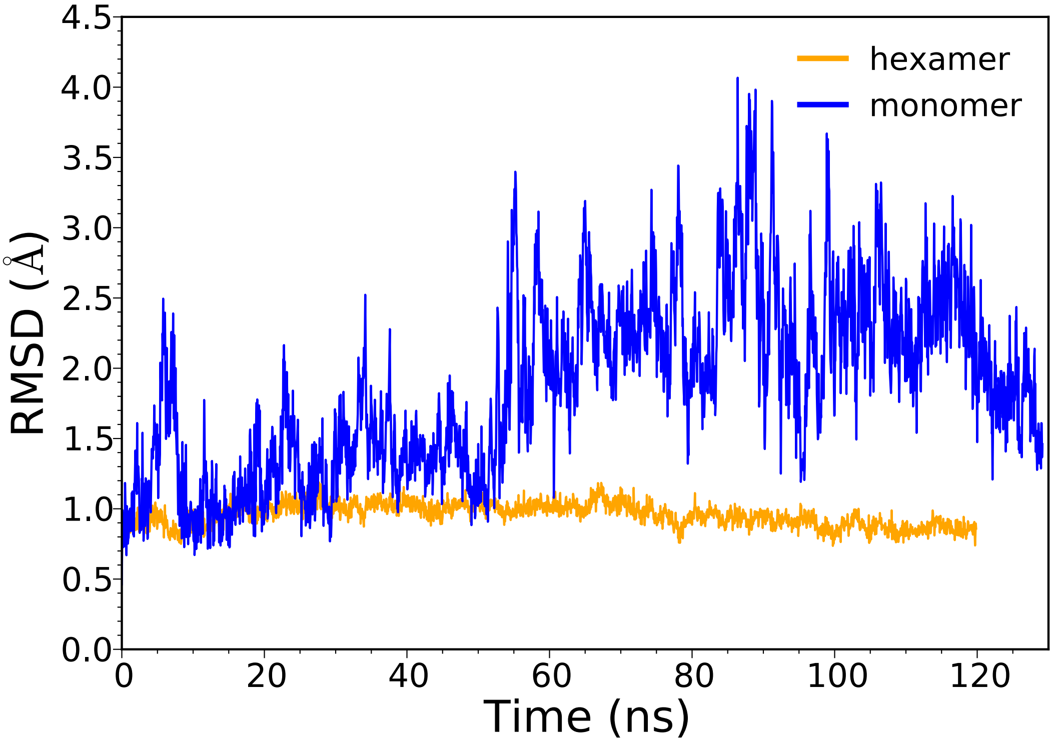

Figure S1. Root mean square deviation from the X-crystal structure (RMSD) of the monomer (blue) and the hexamer (orange), along the molecular dynamics simulation. The RMSD of the monomer stabilises around 2.5 Å, a considerably larger value than that observed for the average monomer in the hexamer (1 Å). The simulation suggests that the monomer native state is stable in solution but slightly deformed (especially at the N-terminus) and more fluctuating relative to the monomer in the hexamer. The simulation of the monomer was performed as described for the hexamer, at the same pressure and temperature, but in a smaller water box containing 24970 TIP3P molecules.

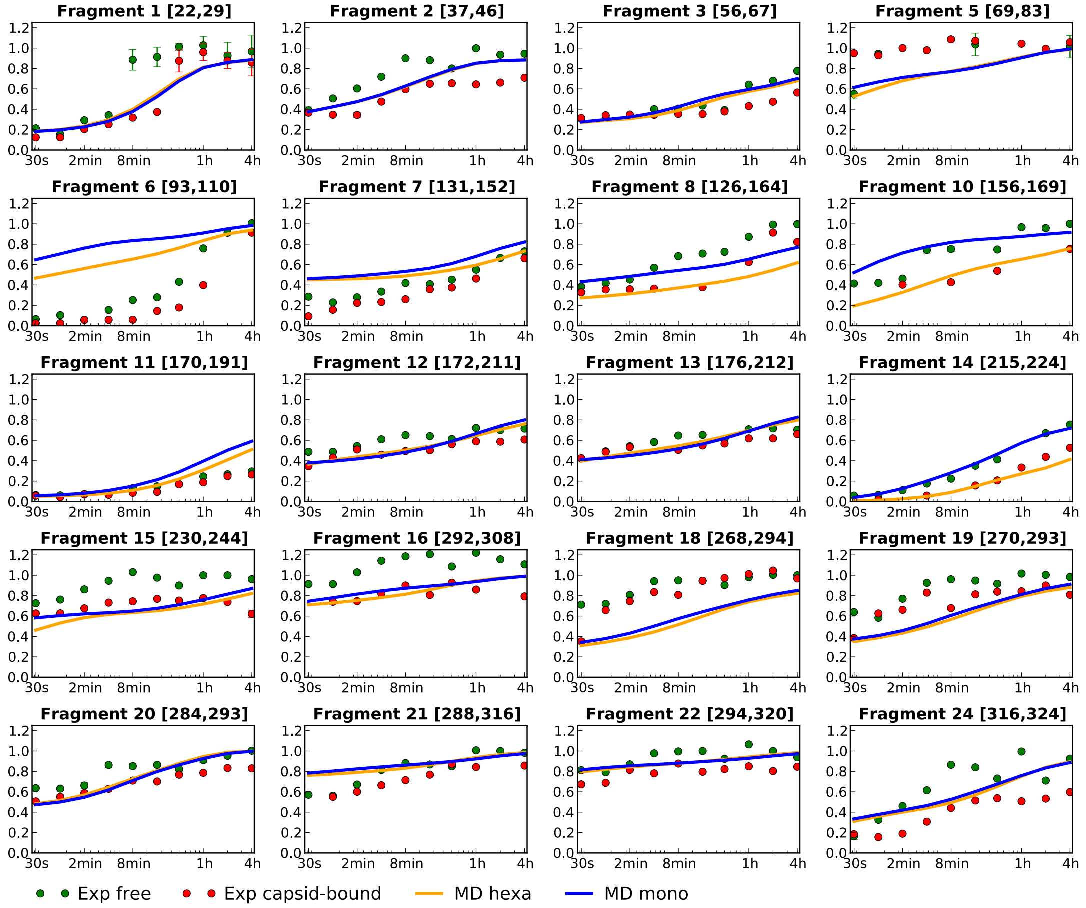

Figure S2. Predicted deuterium fractions (averages over the MD simulation) for the monomer (blue line) and the hexamer (orange line). The experimental deuterium fractions of the free hexamer and the hexamer assembled with the procapsid are shown as green and red dots, respectively. Experimental error bars are shown when larger than the symbols.

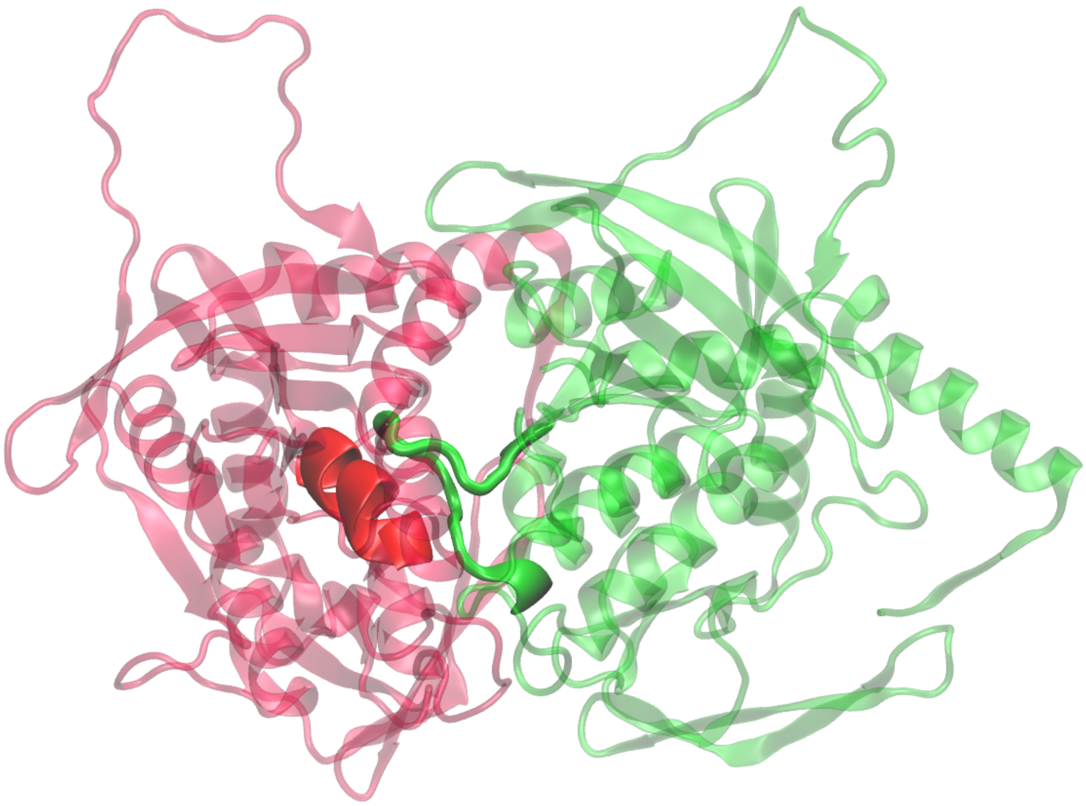

Figure S3. Cartoon representation of the interface between two neighbouring subunits of the hexamer. The fragments 14 and 10 are highlighted in red and green, respectively. For better clarity, the subunits are depicted up-side-down compare to Figures 1 and 4.

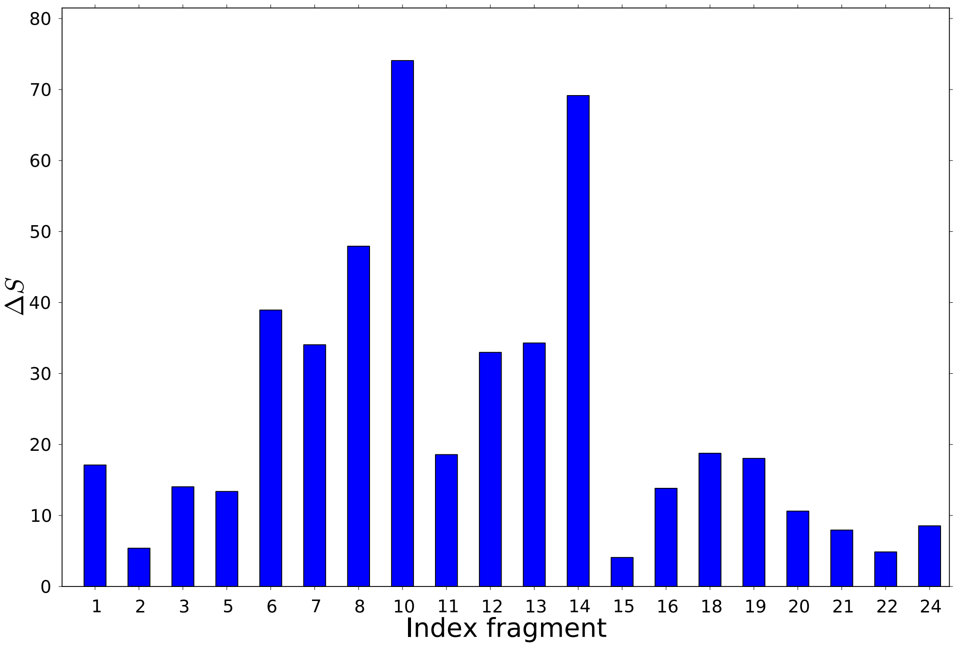

Figure S4. Change of the solvent accessible surface area of each fragment between the monomer and hexamer in the crystal structure.

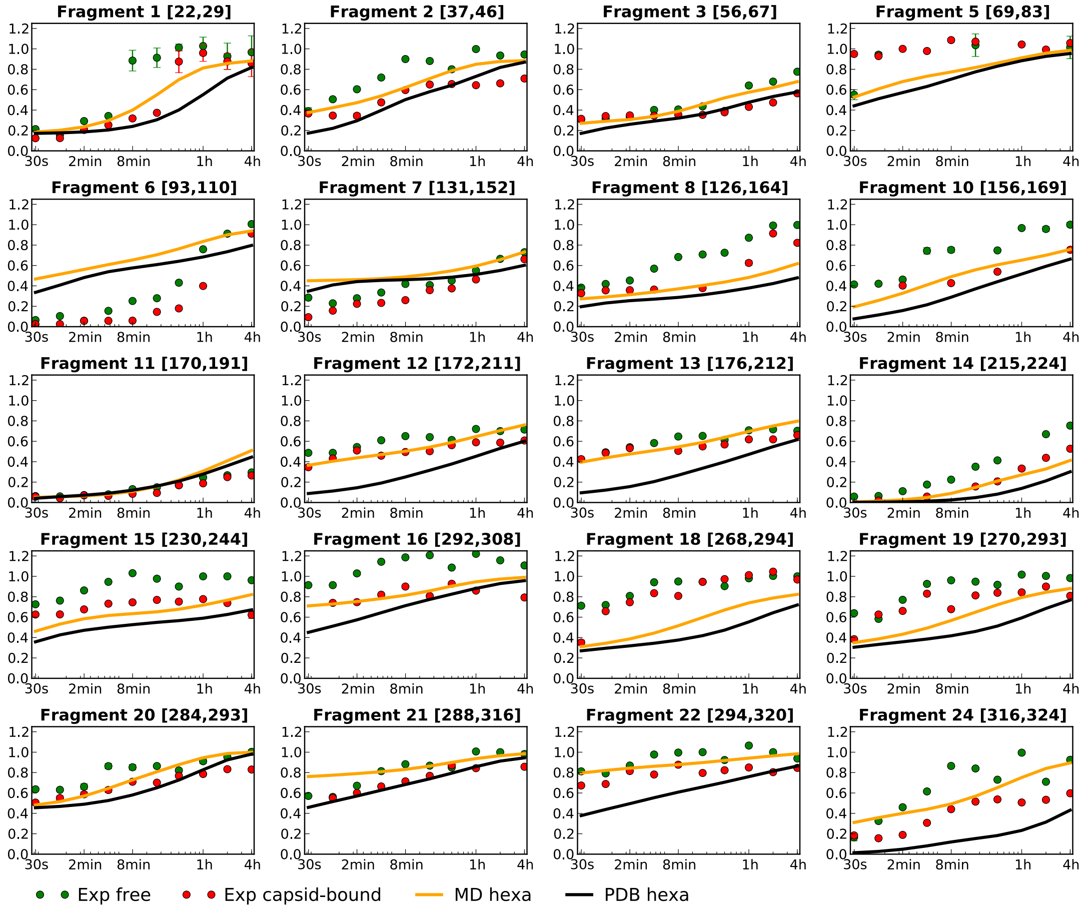

Figure S5. Exchange kinetics of the hexamer with (orange line) or without (black line) dynamics predicted from the MD simulations. The experimental fraction of the free hexamer and the hexamer assembled with the procapsid are represented as green and red dots, respectively. Experimental error bars are shown when larger than the symbols.

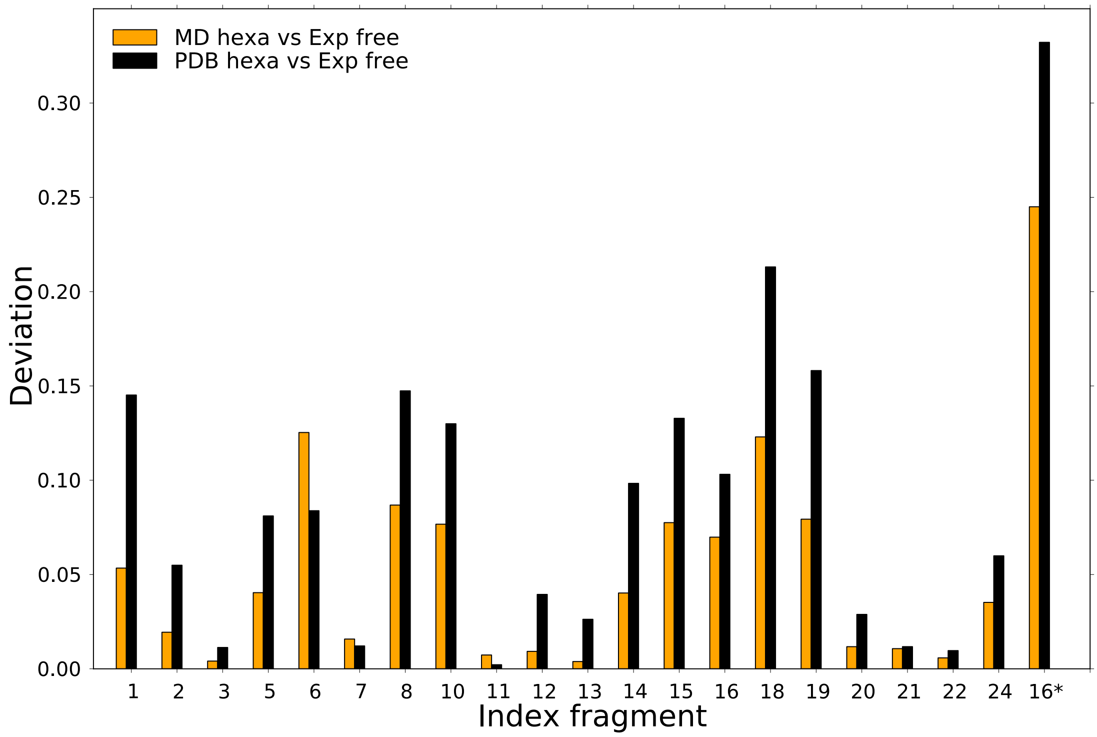

Figure S6. Deviation (χ^2 defined in Eq S2) of the HDX kinetics from the experimental data for the free hexamer as defined by Equation S2, for each fragment. Deviation has been calculated for the crystal structure (black) or the MD simulation of the hexamer (orange). Deviation for the old assignment of the fragment 16 is indicated with *.

List of fragments

Fragment number	Assignment
1	H33-W29
2	L37-V46
3	A56-V67
4	A56-V70
5	Y69-V83
6	Q93-S110
7	K131-K152
8	V126-G164
9	V126-G164
10	V156-F169
11	N170-L191
12	F172-A211
13	I176-F212
14	L215-S224
15	I230-E244
16	S292-S308
16*	I230-L245
17	D239-V246
18	Q268-L294
19	L270-V293
20	L284-V293
21	Y288-I316
22	L294-E320
23	E290-S330
24	I316-V324

Table S1. List of the fragments experimentally probed. The assignment of the fragment 16 proposed here and that reported in Ref [1] are called “new” and “old”, respectively.

Id	Experimental data m/z	z	exp-mass	Old assignment sequence	New assignment sequence	mass	Δm
1	870.3828	1	869.37498	22-29	22-29	869.36676	0.00822
2	1045.5247	1	1044.51688	37-46	36-45	1044.49749	0.01939
					37-46	1044.49749	0.01939
					38-47	1044.49749	0.01939
					98-106	1044.54913	0.03225
					128-138	1044.55635	0.03947
3	687.4032	2	1372.79076	56-67	56-67	1372.78265	0.00811
					57-68	1372.78265	0.00811
4	868.5086	2	1735.00156	56-70	56-70	1734.97805	0.02351
					173-187	1734.95627	0.04529
5	813.4265	2	1624.83736	69-83	69-83	1624.83078	0.00658
					168-181	1624.79189	0.04547
6	963.9651	2	1925.91456	93-110	93-110	1925.94174	0.02718
7	726.413	3	2176.21554	131-152	131-152	2176.19635	0.01919
8	785.6373	5	3923.1474	126-164	126-164	3923.12678	0.02062
9	981.7956	4	3923.15112	126-164	126-164	3923.12678	0.02434
10	801.3862	2	1600.75676	156-169	156-169	1600.75214	0.00462
					160-173	1600.74091	0.01585
					38-53	1600.78315	0.02639
11	842.1326	3	2523.37434	170-191	170-191	2523.36311	0.01123
12	1020.807	4	4079.19672	172-211	172-211	4079.16974	0.02698
					173-212	4079.16974	0.02698
13	938.525	4	3750.06872	176-212	176-212	3750.04745	0.02127
14	935.4548	1	934.44698	215-224	215-224	934.44296	0.00402
					117-125	934.40792	0.03906
					206-214	934.4872	0.04022
					290-297	934.48721	0.04023
15	808.4156	2	1614.81556	230-244	230-244	1614.81005	0.00551
					231-245	1614.81005	0.00551
					238-251	1614.85766	0.0421
16	864.9627	2	1727.90976	230-245	292-308	1727.91659	0.00683
					140-156	1727.92058	0.01082
					230-245	1727.89411	0.01565
17	930.5123	1	929.50448	239-246	239-246	929.50693	0.00245
					312-319	929.51818	0.0137
					81-89	929.48179	0.02269
					142-151	929.48177	0.02271
					305-313	929.52941	0.02493
18	756.905	4	3023.58872	268-294	268-294	3023.57881	0.00991
					257-283	3023.56756	0.02116
					258-284	3023.56756	0.02116
19	671.8521	4	2683.37712	270-293	269-292	2683.36776	0.00936
					270-293	2683.36776	0.00936
					271-294	2683.36776	0.00936
					272-295	2683.36776	0.00936
					42-67	2683.41802	0.0409
20	1120.5242	1	1119.51638	284-293	284-293	1119.51964	0.00326
					285-294	1119.51964	0.00326
					258-267	1119.50839	0.00799
					17-26	1119.4985	0.01788
					55-64	1119.55601	0.03963
					280-289	1119.55603	0.03965
					106-116	1119.5634	0.04702
21	1009.2095	3	3024.60504	288-316	288-316	3024.5992	0.00584
21					292-320	3024.62033	0.01529
22	710.6451	4	2838.54912	294-320	294-320	2838.51989	0.02923
22					295-321	2838.51989	0.02923
23	1112.3603	4	4445.40992	290-330	290-330	4445.39897	0.01095
24	1046.5303	1	1045.52248	316-324	315-323	1045.52913	0.00665
					316-324	1045.52913	0.00665
					317-325	1045.52913	0.00665
					263-271	1045.508	0.01448

Table S2. Assignment for each fragment. In each column is indicated the index of the fragment, the experimental monoisotopic mass of the fragment, the number of charge, the previous assignment, the first residue of the new assignment, the last residue of the new assignment, the

monoisotopic mass of the new fragment, the absolute difference of mass between the experimental and predicted mass of the new fragment, respectively.

1. Lísal, J., et al., *Interaction of packaging motor with the polymerase complex of dsRNA bacteriophage.* Virology, 2006. **351**(1): p. 73-79.
