# Supplementary material for: Functional Dynamics of Hexameric Helicase Probed by Hydrogen Exchange and Simulation
Source: Biophys J. 2014 Aug 19;107(4):983–90. doi: 10.1016/j.bpj.2014.06.039 (PMC4142241; doi:10.1016/j.bpj.2014.06.039)
Supplement: Document S2. Article plus Supporting Material [file mmc2.pdf]

Article

Functional Dynamics of Hexameric Helicase Probed by Hydrogen Exchange and Simulation

Gaël Radou,[1] Frauke N. Dreyer,[1] Roman Tuma,[1,*] and Emanuele Paci[1,*]
[1]Astbury Centre for Structural Molecular Biology, University of Leeds, Leeds, United Kingdom

ABSTRACT The biological function of large macromolecular assemblies depends on their structure and their dynamics over a broad range of timescales; for this reason, it is a significant challenge to investigate these assemblies using conventional experimental techniques. One of the most promising experimental techniques is hydrogen-deuterium exchange detected by mass spectrometry. Here, we describe to our knowledge a new computational method for quantitative interpretation of deuterium exchange kinetics and apply it to a hexameric viral helicase P4 that unwinds and translocates RNA into a virus capsid at the expense of ATP hydrolysis. Room-temperature dynamics probed by a hundred nanoseconds of all-atom molecular dynamics simulations is sufficient to predict the exchange kinetics of most sequence fragments and provide a residue-level interpretation of the low-resolution experimental results. The strategy presented here is also a valuable tool to validate experimental data, e.g., assignments, and to probe mechanisms that cannot be observed by x-ray crystallography, or that occur over timescales longer than those that can be realistically simulated, such as the opening of the hexameric ring.

INTRODUCTION

Proteins are highly dynamic molecular entities (1), and their conformational variability is essential to their function (2). This is particularly the case for macromolecular complexes that play essential roles in the cell, such as molecular motors (3,4). A powerful technique to investigate the dynamics of large proteins and their complexes is hydrogen-deuterium exchange probed by mass spectrometry (HDX-MS) (5,6). This method is based on the spontaneous exchange of the amide hydrogens of the protein with deuterium from solvent containing deuterium oxide (^2H_2O), and it has been extensively used to investigate protein folding (7–10). Key to interpreting HDX kinetics is the fact that exchange occurs faster for amides that are solvent-exposed and not involved in hydrogen bonds.

Deuterium incorporation has been measured using NMR with residue-level resolution (11) for small proteins; for larger proteins and assemblies, detection of HDX by high-resolution mass spectrometry (MS) has emerged as an alternative (12,13). HDX-MS relies on the measurable difference in mass between the deuterated and nondeuterated polypeptide chain. Fragmentation of the protein by proteolysis at low pH and low temperature (i.e., conditions that reduce exchange and thus preserve the isotopic pattern even under nonnative conditions, with the residual forward and back exchange readily corrected for (14,15)) allows determination of region-specific exchange (usually covering 10–20 amino acid segments) (16). The deuterium incorporated into the side-chain groups is rapidly back-exchanged. As a consequence, HDX-MS is only sensitive to the backbone amide exchange. Recent advances in MS (e.g., electron capture dissociation (17)) and development of in-line proteolysis (18) suggest that HDX tandem mass spectrometry can be used to measure hydrogen exchange at single-residue resolution. However, the required uniform coverage and resolution of isotopic envelopes may be hard to achieve for larger proteins and multiprotein assemblies (19). Monitoring incorporation of deuterium for each fragment over time yields exchange kinetics. This contains information about local and global stability averaged over all amide NH groups within the fragment. HDX-MS data are usually limited to qualitative analysis, e.g., by mapping the apparent rate of exchange of different fragments on the available structure and comparing directly the kinetics of the same fragments under different conditions (15,20,21), although computational methods have been proposed to predict HDX of proteins from structure (22–26).

Hexameric packaging motors (P4 proteins (Fig. 1 *A*)) from cystoviruses φ6, φ8, φ12, and φ13 are responsible for genome translocation into preformed capsids using energy from ATP hydrolysis (27). These proteins have been characterized extensively, and many high-resolution structures in different conformational states are available (28–30), making them an excellent model system for the related SF4 helicases (31). HDX-MS kinetics have been obtained for a free hexamer and capsid-bound φ12 P4 and qualitatively interpreted previously (20).

The φ12 P4 subunit is composed of three regions: the N-terminal apical domain, the conserved RecA-like ATPase

Submitted March 31, 2014, and accepted for publication June 24, 2014.

*Correspondence: e.paci@leeds.ac.uk or r.tuma@leeds.ac.uk

This is an open access article under the CC BY license (http://creativecommons.org/licenses/by/3.0/).

Editor: Michael Feig.

© 2014 The Authors

0006-3495/14/08/0983/8 $2.00

http://dx.doi.org/10.1016/j.bpj.2014.06.039

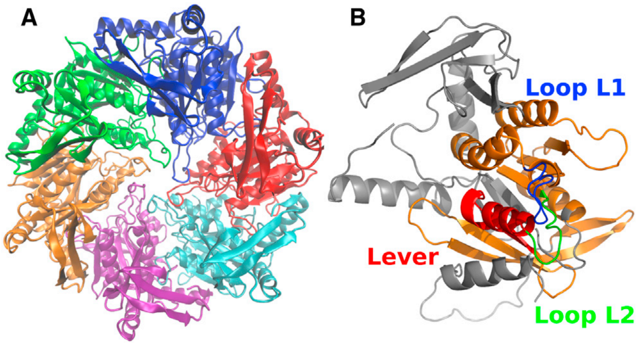

FIGURE 1 Structure of φ12 P4. (*A*) X-ray crystal structure of the hexamer φ12 P4 in the apo state, with the subunits shown in different colors. (*B*) Elements located in the channel and interacting with RNA (Loops L1 (*blue*) and L2 (*green*)). The conserved RecA-like nucleotide-binding domain is colored orange and the lever red. In the depicted monomeric structure shown, the lever is in a down state. In the proposed mechanism, the lever is locked in an up conformation in the ATP-bound state and moves to the down configuration as a result of hydrolysis and phosphate release (28). To see this figure in color, go online.

core, and the C-terminal extension (Fig. 1 *B*). The C-terminal extension (residues 290–331) is essential for the binding of the hexamer to the capsid (20,27). Loops L1 (residues 196–206, partially disordered) and L2 (residues 233–238) protrude into the central channel where they contact RNA during translocation (21,32). The loop L2 together with helix α6 constitutes a moving lever that effects the translocation power stroke (29).

In this article, we estimate the deuterium fractions of any chain fragment of the packaging motor P4 from bacteriophage φ12 from molecular dynamic simulations of the native state of the P4 hexamer and monomer. We show that an ~100 ns simulation is sufficient to predict (with some instructive exceptions) the experimental exchange kinetics for times ranging from seconds to hours. Thus, the simulation provides a high-resolution representation of the microscopic structures and dynamics responsible for the HDX over several orders of magnitude in time, which is validated by the experiment. The method we propose also turns out to be a powerful tool to validate the assignment of the fragments, to assess the structure of modeled regions missing from the crystal structure and to probe conformational variability that cannot be observed by x-ray crystallography.

THEORY AND METHODS

At neutral pH, the exchange is fast for solvent-exposed amides whereas hydrogen bonding, e.g., within helices or β-sheets, slows it down. When fully exposed, the exchange kinetics of the amide is governed by an intrinsic rate, k_{int}, that depends on the temperature, solution pH, and side chains of the two neighboring residues (33). Within a folded protein, the exchange of amide hydrogen requires local opening of the structure and can be approximated as a two-step process (34):

$$NH_{cl} \overset{k_{cl}/k_{op}}{\longleftrightarrow} NH_{op} \overset{k_{int}}{\longrightarrow} ND_{op}, \qquad (1)$$

where k_{cl} and k_{op} are the local closing and opening rates. The observed deuterium uptake rate, k_{obs}, can be expressed as

$$k_{obs} = \frac{k_{int}k_{op}}{k_{int} + k_{op} + k_{cl}}. \qquad (2)$$

Two limiting regimes, called EX1 and EX2, are invoked in interpreting HDX kinetics of proteins. For both regimes, the protein is considered to be in native conditions, i.e., $k_{cl} \gg k_{op}$. In the EX1 limit, $k_{int} \gg k_{cl}$ implies that the amide exchanges as soon as it becomes exposed to solvent, i.e., $k_{obs} = k_{op}$. In this regime, the exchange is limited by slow conformational changes that are usually associated with global unfolding (35) or cooperative changes in quaternary structure (21). This regime is readily discerned by a bimodal pattern of isotopic distribution in mass spectra (undeuterated and deuterated species) and by pH independence. In the EX2 limit, $k_{cl} \gg k_{int}$,

$$k_{obs} = \frac{k_{int}}{P}, \qquad (3)$$

where $P = k_{cl}/k_{op}$ is a protection factor for the particular amide. The EX2 limit governs exchange under native conditions and is sensitive to local stability. In the EX2 regime, the kinetics is sensitive to pH (through k_{int}) and the corresponding isotopic envelope moves progressively to the fully deuterated limit.

Computational methods are based on estimation of protection factors either by calculating the difference of free energy between the open and closed states, $\Delta G = RT \ln P$ (24,36,37) or by relating the protection factor to the local environment of the residue (10,38,39). Solvent accessibility is generally used to predict the exchange competence of a residue. Although a strong correlation exists between protection factors and solvent accessibility, many residues located at the protein surface (i.e., totally solvent-exposed) exhibit exchange rates much slower than their intrinsic rates (38,40). Indeed, it is now well established that exchange of amide hydrogens also requires the breaking of hydrogen bonds formed with the side chains or the protein backbone (22). In the EX2 regime, the protection factor of an amide hydrogen of residue *i* can be approximately estimated from the structure of the protein using the phenomenological equation (10,39)

$$\ln P_i^{sim}(X) = \beta_c N_i^c(X) + \beta_h N_i^h(X), \qquad (4)$$

where *X* is a particular conformation of the protein and $N_c(X)$ and $N_h(X)$ are the number of contacts between nonhydrogen atoms and the number of hydrogen bonds to the amide hydrogen, respectively. In this approximation, the hydrogen exchange rate is governed primarily by the burial of the amide within the hydrophobic core or subunit interface and by participation in secondary structure. The phenomenological approximation in Eq. 4 can be used to predict or attempt interpretation of experimental HDX data from a single protein structure. In doing so, however, one neglects thermal fluctuations and conformational heterogeneity that contribute to the exchange (41,42). Assuming the validity of Eq. 4, protection factors should then be estimated as an ensemble average, which can be done through an equilibrium molecular dynamics simulation.

The HDX-MS data of φ12 P4 hexamer in the apo state have been previously collected at 298 K and pH 7.9 (20); experiments were conducted on the free hexamer in solution and on the capsid-bound form of φ12 P4 (20). For both experiments exchange kinetics had been reported for 24 fragments; in all cases, kinetics exhibited an EX2 regime pattern, a necessary condition for the approximation in Eq. 4. Four fragments, 4, 9, 17, and 23, were discarded from the original data set (Table S1), leaving 20 fragments that cover 70% of the protein sequence. The discarded fragments were either overlapping with another (fragments 4, 9, and 23) or affected by a large experimental uncertainty (fragment 17).

Simulations of the φ12 P4 hexamer and monomer in the apo state were performed with NAMD using the CHARMM36 force field; 77,349 TIP3P water molecules were included to ensure that at least 10 Å separated

periodic images of the proteins, as well as 235 Na ions and 205 Cl ions to set the ion concentration at 0.15 M. The crystal structure of the apo state φ12 P4 (30) (PDB access code 4BLR) was used as initial conformation. The missing residues (196–206, 236–241, 299–312, 329–331) were modeled using MODELER (43). Simulations were performed at 298 K and atmospheric pressure. Periodic boundary conditions were applied and long-range electrostatic interactions were calculated with the particle mesh Ewald method, with a cutoff of 12 Å and grid spacing of 1 Å. Neighbor-atom lists were constructed that included all atoms <14 Å away from a given atom. A 2 fs time step was used and conformations were saved every 500 time steps (1 ps). The production runs were 100 ns long and were preceded by a 20 ns equilibration during which temperature was increased from 0 to 298 K by 20 K increments every 500 ps.

The phenomenological Eq. 4 depends on parameters β_c and β_h. We used the values $\beta_c = 0.35$ and $\beta_h = 2$, previously shown to provide the best prediction for a set of seven proteins for which residue-specific data were available (39). To calculate N_h, a hydrogen bond was considered as formed when the distance between the amide hydrogen and the acceptor oxygen was <2.4 Å. N_c was measured as the number of heavy atoms (nonhydrogen atoms) <6.5 Å away from the amide nitrogen. If residue *i* contains an amide hydrogen, the protection factor, P_i, is defined as

$$P_i = \frac{k^i_{int}}{k^i_{obs}}, \tag{5}$$

where k^i_{int} and k^i_{obs} are the intrinsic and observed exchange rates, respectively, of residue *i*. Thus, the deuterium fraction of residue *i* at time *t* is

$$D_i(t) = 1 - e^{-\left(k^i_{int}/P_i\right)t}. \tag{6}$$

The protection factor of each residue was calculated as in Eq. 4, using CHARMM (44). The intrinsic exchange rates were estimated as described in Bai et al. (33). Thus, the deuterium fraction, $D^{sim}_j(t)$, of fragment *j* at time *t* was

$$D^{sim}_j(t) = \frac{1}{n_j}\sum_{i=1}^{n_j} D_i(t) = \frac{1}{n_j}\sum_{i=m_j}^{m_j+n_j-1}\left(1 - e^{-\left(k^i_{int}/P^{sim}_i\right)t}\right), \tag{7}$$

where n_j and m_j are the number of amide hydrogens and the index of the first residue in fragment *j*, respectively. Solvent-accessible surface was calculated using NACCESS (http://www.bioinf.manchester.ac.uk/naccess) with a probe radius of 1.4 Å.

RESULTS

Protection factors for each residue of the apo φ12 P4 hexamer were calculated using Eq. 4 and averaged over a 100 ns MD trajectory at room temperature (Fig. 2 *A*). Protection factors are generally smaller for residues exposed to solvent (Fig. 2 *B*) and for residues located in particularly flexible regions, i.e., characterized by larger positional fluctuations (Fig. 2 *C*). This is the case of loops A76–S80, L1, L2, and part of the C-terminus (S299–I312), which have a large root mean-square fluctuation consistent with them being absent from the crystal structure or having a large B factor. Protection factors obtained from the crystal structure are systematically higher than those obtained from the simulation (Fig. 2 *A*), particularly in regions exhibiting higher fluctuations. This reflects the mechanism of EX2 exchange in which local conformational fluctuations mediate instantaneous solvent accessibility.

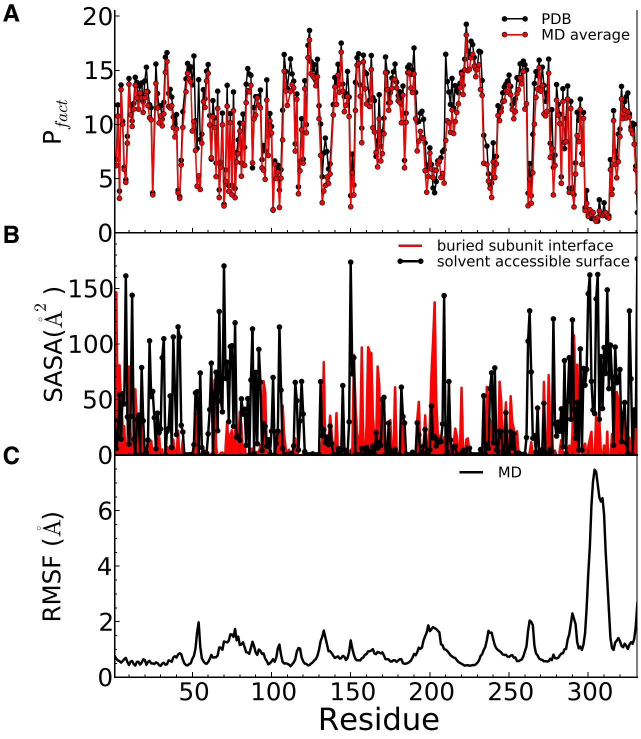

FIGURE 2 Structural and dynamical characteristics of residues. (*A*) Protection factors calculated for the crystal structure of the hexamer of φ12 P4 (*black*) and time-averaged over a 100 ns simulation (*red*). In both cases, they represent the average over all monomers within the hexamer. (*B*) Solvent-accessible surface calculated from the crystal structure for the hexamer of φ12 P4 (*black line*) and variation of solvent-accessible surface area between the monomer and the hexamer (*red line*). The latter corresponds to the surface buried upon oligomerization and is shown to highlight the interfaces between monomers. (*C*) Root mean-square fluctuation of the hexamer from its structure averaged over the 100 ns simulation. To see this figure in color, go online.

The time-dependent deuteration of each fragment $D(t)$ was calculated using the protection factors calculated for each residue and Eq. 7 Fig. 3 *A* illustrates $D(t)$ for selected fragments that have been analyzed by MS but over a broader time interval than accessible experimentally with manual mixing (Fig. 3 *A*, *dashed vertical line*). It is clear that $D(t)$ also provides valuable information over shorter timescales that require rapid mixing and quenching.

Direct comparison between calculated and experimental $D(t)$ for the 20 nonredundant fragments from Lisal et al. (20) is shown in Fig. S2. In Fig. 3 *B* are plotted the $D(t)$ values calculated from simulation (*y* axis) against the experimental data (*x* axis) for the free hexamer for each fragment and time point for which experimental data are available. Although points concentrate around the diagonal, the prediction is rather poor for a few fragments.

One possible reason for poor prediction is incorrect assignment, which may result from interpreting tandem mass spectra of a complex mixture of primary ions. The assignment of each fragment has thus been checked. Interestingly, the monoisotopic mass of fragment 16, originally

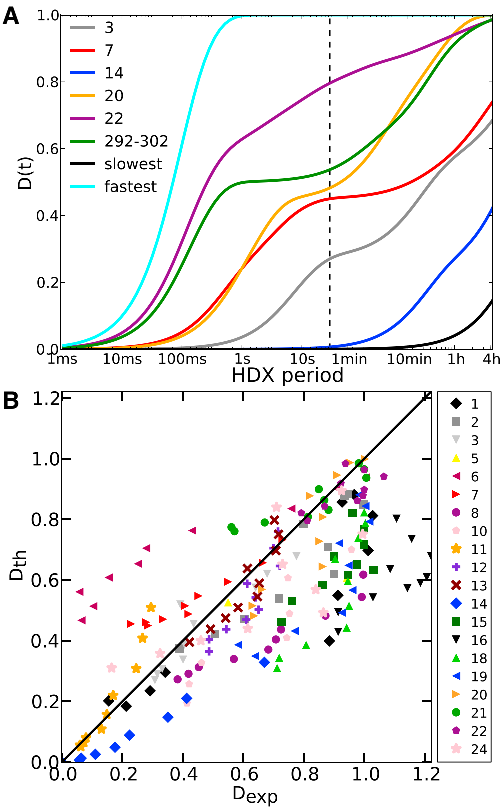

FIGURE 3 Comparison between deuterium-fraction predictions using the hexameric state and experimental data. (*A*) Estimated deuterium fractions, $D(t)$, for selected fragments (see Table S2 for assignments), including three that were not probed experimentally but represent the fastest- and slowest-exchanging 11-residue fragments (residues 301–311 and 220–230, respectively) and fragment 292–302, which was chosen to highlight how its kinetics are similar to that of fragment 20 at long timescales but different at shorter ones. The vertical dashed line designates the fastest time experimentally measurable with manual mixing. (*B*) Estimated versus experimental deuterium fractions, $D(t)$ (including the time points $t = 30$ s, 1 min, 2 min, 4 min, 8 min, 15 min, 30 min, 1 h, 2 h, and 4 h), of all fragments of φ12 P4 (free in solution). Each fragment is represented by a different symbol/color. The diagonal line represents a perfect match. To see this figure in color, go online.

assigned to residues 230–245, matches better that of a fragment encompassing residues 292–308 (Table S2). The predicted kinetics of the newly assigned fragment is in excellent agreement with the experimental kinetics, suggesting that the correct assignment should have been 292–308 (Fig. 4 *A*). However, no better assignment was found for the other fragments that exhibit a discrepancy between experiment and prediction.

For fragment 14 (Fig. 4 *B*), we predicted an exchange slower than that of the experimental one for free hexamers but in excellent agreement with that measured for the capsid-bound hexamer. Since this fragment is located at the subunit interface in the hexamer, it is conceivable that the faster experimental exchange is related to ring opening and its slowing is consistent with stabilization of the hexamer by interactions with the capsid. These additional

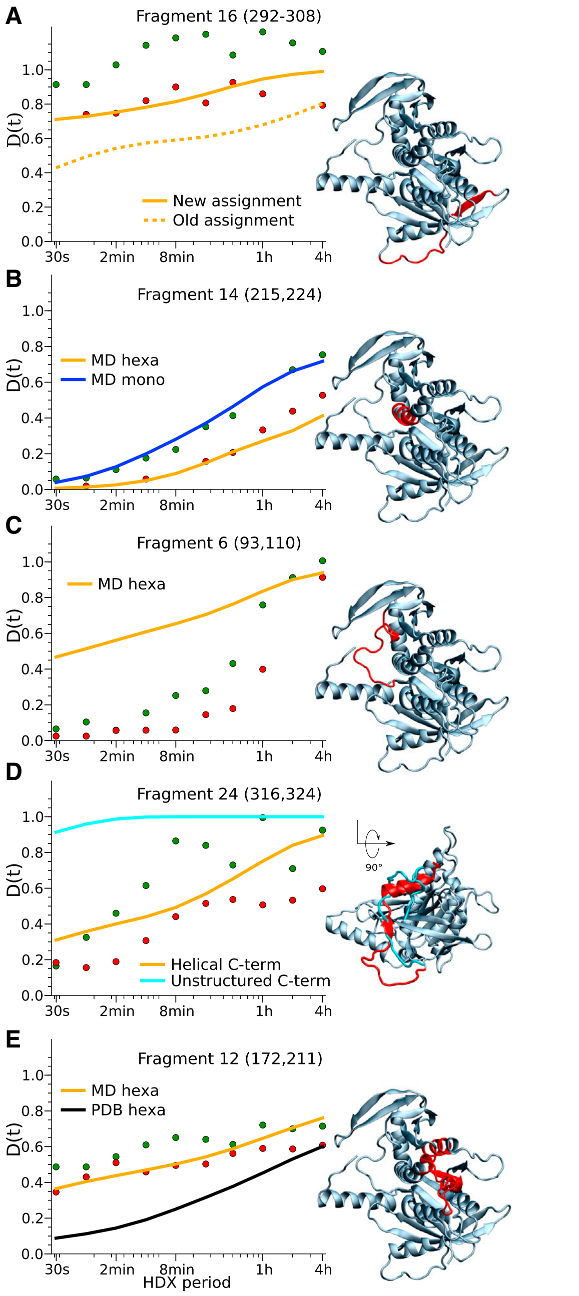

FIGURE 4 HDX kinetics of five relevant fragments: 16 (S292–S308) (*A*), 14 (L215–S224) (*B*), 6 (Q93–S110) (*C*), 24 (I316–V324) (*D*), and 12 (172–211) (*E*). The experimental exchange kinetics of the free and capsid-bound hexamers are shown as green and red circles, respectively. In the structures shown at right, each fragment is highlighted in red within the structure of one subunit of φ12 P4. In *D*, the whole C-terminal domain (K300–N331) is highlighted instead of only fragment 24, and the view is rotated 90° with respect to those of *A*–*C* and *E*. The modeled C-terminal domain is highlighted in cyan. To see this figure in color, go online.

interactions prevent large conformational changes such as dissociation of subunit interfaces, therefore keeping the fragments localized at the interface buried. We thus formulated the hypothesis that the free form consists of a mixture of hexamers and open hexamers or lower-order assemblies,

including monomers. We simulated a single solvated monomer for 100 ns (for details, see caption for Fig. S1 in the Supporting Material) and estimated the exchange kinetics of each fragment (Fig. S2). Regions at the monomer-monomer interface or within the channel in the hexameric structure are exposed to solvent in the monomeric form, and their exchange is predicted to be faster than in the hexamer, whereas exchange kinetics remains unchanged for fragments located farther from the interface (Figs. S3 and S4). Comparing experimental HDX kinetics of the hexamer free in solution with that of the capsid-bound hexamer indicates that exchange is significantly faster also for fragment 10, which is completely buried in the monomer-monomer interface like fragment 14. The crystal structure of φ12 P4 reveals that fragments 10 and 14 are adjacent at the core of the monomer-monomer interface, such that fragment 10 is exposed to solvent if and only if fragment 14 is exposed as well (Fig. S3). Since fragment 14 is helical, its secondary structure further limits the hydrogen exchange process even when it is exposed during ring opening. In contrast, fragment 10 lacks regular secondary structure and rapidly exchanges when exposed to solvent.

For fragment 6 (Fig. 4 *C*), we predicted faster exchange than that measured experimentally. Fragment 6 encompasses residues 93–110, which are located in a loop close to the monomer-monomer interface. In simulations, the loop fluctuates and remains solvent-exposed, as it is the case in the crystal structure, leading to the fast exchange kinetics prediction. One explanation is that in solution, the loop may adopt a form more stable than it appears in the crystal structure and that this alternative conformation would be attained on a longer timescale than that of the current simulation.

It is instructive here to mention the case of fragment 24, which constitutes the C-terminus. As shown in Fig. 4 *D*, despite a quite large dispersion of the experimental results, the trend is well predicted by the native simulation. The C-terminus was only partially resolved (residues 301–331 disordered) in the previous crystal structure (29) (PDB access code 1W4C). We initially performed the simulation described in Theory and Methods starting from 1W4C and modeling the C-terminal region as a flexible region (43). As a result, the C-terminus was quite dynamic and explored different conformations, resulting in a large overestimation of the fraction of deuterium exchanged by fragment 24 at all times (at $t = 30$ s, 90% is exchanged already compared to the experimental value of ~20% for both the free and capsid-bound hexamer), likely because the region was not correctly modeled. This finding highlights the possible utility of the method to validate structural models.

For fragments 18 and 19, we predicted slower exchange than measured experimentally. Ring opening could not explain this mismatch, since these fragments are not localized at the interface of two subunits. Hence, the kinetics of these two fragments suggests a local conformational change, which has not been captured over the 100 ns simulation. The mismatch is less pronounced for fragment 19, whereas the kinetics of fragment 20 is accurately predicted, suggesting that the conformational change occurs in the region between residues 268 and 284, which encompasses one side of the ATP binding site and includes the highly mobile arginine finger 279. This region has been shown to be highly dynamic and responds to ATP and RNA binding in φ8 P4 (21,30).

We have seen above that a native-state ensemble as sampled by a 100 ns room-temperature simulation reproduces the experimentally probed HDX occurring on timescales ranging from 30 s to hours, except in specific regions for which we have to assume that conformational changes and large-scale fluctuations not sampled by the 100 ns simulation may occur. In fact, a relatively fair prediction could also be obtained by neglecting the native-state dynamics altogether and estimating the protection factors and $D(t)$ from Eqs. 4–7 using the crystal-structure coordinates. Indeed, exchange would be predicted to be systematically slower (Figs. 4 *E* and 5), and this could be corrected by refitting the parameters β_c and/or β_h. However, the overall discrepancy between calculated and experimental $D(t)$, with all the caveats discussed above about the two different sets of experimental $D(t)$, would be larger. The importance of accounting for dynamics by estimating $D(t)$ using protection factors calculated as ensemble averages is particularly evident for a few fragments, such as fragment 12, for which the fraction of deuterium exchange is seriously underestimated if calculated from the crystal structure alone (Fig. 4 *E*). This is also the case for other fragments, such as 13, 22, and 24, that encompass a loop (Figs. S5 and S6).

The importance of estimating HDX as an average over a realistic ensemble of the relevant states is clear from the evolution of protection factors along the trajectory; as shown in Fig. 5 for four fragments, the instantaneous

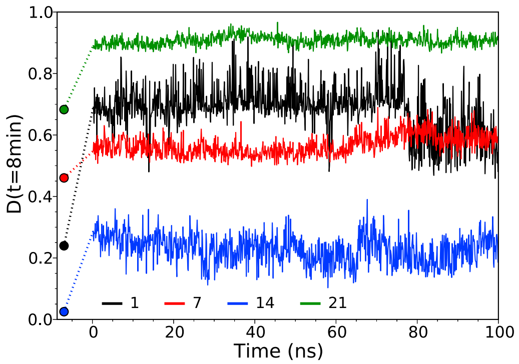

FIGURE 5 Importance of interpreting HDX data through an ensemble of structures. Time series of the deuterium fractions for fragments 1, 7, 14, and 21 at $t = 8$ min ($D(t = 8\text{min})$) were calculated for structures along the trajectory for a single subunit within the hexamer (i.e., without averaging over the six subunits). The circles show the corresponding initial values obtained from the crystal structures. To see this figure in color, go online.

estimated deuterium content varies significantly along the trajectory. Particularly interesting is the case of fragment 14, where the fraction of deuterium exchanged at t = 8 min varies between 0.10 and 0.40 along the trajectory, with an average of 0.23, in excellent agreement with the experimental value (0.22), but considerably different from the value obtained from the crystal structure alone (0.02).

DISCUSSION

We devised and tested a method that is based on detailed atomistic simulation to sample the native bound state for a large complex, such as a hexameric helicase, and allows prediction of HDX, and facilitates direct, quantitative comparison with experimental data. The results show that native-state dynamics is necessary and sufficient to predict, with some instructive exceptions, the HDX kinetics occurring over a timescale extending over six orders of magnitude. The method's central assumption is that the protection factor of individual residues can be estimated as an ensemble average of a function of the atomic coordinates of the protein, and that such a function can be empirically approximated as the sum of two terms, one proportional to the number of hydrogen bonds and the other to the number of contacts with neighboring residues. Such an approximation has been previously proposed and shown to provide a relatively good prediction of the protection factors measured by NMR for small proteins (39). Here, we use the same approximation to estimate, as a function of time, the fraction of deuterium exchanged by fragments of a large protein and to directly compare the predictions with HDX/MS measurements. The overall good agreement with experiment confirms the validity of the central assumption of the method. The second assumption is that HD exchange on timescales from milliseconds to hours depends on the native-state dynamics and that the ~100 ns trajectory accurately samples the ensemble of structures representing the bound native state.

Qualitative agreement with the experimental results is important because it validates the aforementioned assumptions behind the theoretical estimation, namely that the protection factors can be estimated by calculating burial from solvent and hydrogen bonding of individual amides and averaging these quantities over the equilibrium simulation trajectory. This provides atomic resolution of the underlying dynamics and structural variability that is captured in the experiment over times ranging from seconds to hours.

This work has implications for refining HDX-MS methodology and for high-resolution structure validation. The first is illustrated by the discrepancy between the prediction and experiment for fragment 16 (Fig. 4 *A*), which was due to an incorrect assignment, an issue particularly important for larger, more complex assemblies. The other discrepancy reflected the wrong assumption about disorder in the C-terminal region based on the absence of electron density in the original crystal structures. Simulations that employed the more recent, higher-resolution structure, in which the C-terminal region is helical, led to a slower exchange kinetics that in turn is in excellent agreement with the experiment (Fig. 4 *D*). This demonstrates that the quantitative prediction can be used as a quality check in HDX-MS experiments and also can complement x-ray crystallography in assessing modeled structures that are otherwise not resolved in the electron density.

The method also provides additional insights into the mechanism of the packaging motor. A quantitative comparison between the experimental and predicted kinetics for the free and capsid-bound hexamer, respectively, demonstrates that the free hexamer exists in a rapid equilibrium between closed and open conformation (Fig. 4 *B*). On the other hand, the procapsid-bound hexamer matches well the intact hexamer prediction (Fig. S2, *fragment 14*) and thus adopts the closed conformation. Since the ring opening is required for RNA loading into the hexamer, it has been proposed for the φ8 bacteriophage that the capsid-bound P4 is in the open conformation (45). This is clearly not the case for φ12 P4.

Fig. 6 illustrates another benefit that the prediction brings to interpretation of HDX-MS. Although in principle possible, especially with the new ECD technology, residue-specific information is seldom obtained for large proteins and their complexes. In cases where there is a good match between the fragment-specific experimental data, one can assume this reflects the overall good prediction on the residue level and use the prediction to further interpret the observations. For example, the conserved P-loop (Walker A or H1 helicase motif involved in Mg and ATP coordination) fragment exhibits a biphasic kinetics (Fig. S2, *fragment 8*) leading to an intermediate overall exchange rate (Fig. 6 *A*), whereas the predictions uncover great variations (Fig. 6 *B*). Contrary

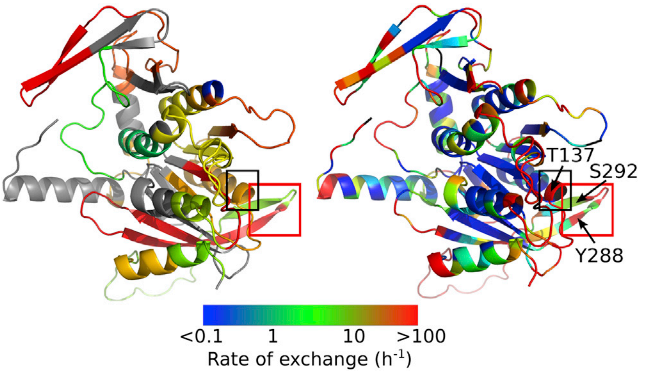

FIGURE 6 Comparison between the experimental fragment-specific apparent exchange rates (*left*) and the residue-specific predicted rates (*right*). The apparent rates in *A* were calculated as described previously (20). The black box delineates the P-loop and the red box highlights the nucleobase-binding hairpin. In *B*, residues without amide hydrogen are in black (e.g. tip of N-terminal), whereas black arrows point to essential residues for translocation that are discussed in the concluding discussion part. To see this figure in color, go online.

to expectations, the tip of the helix, which encompasses the conserved Thr^{137}, is more flexible than certain parts of the loop upstream. As expected, the rest of the downstream helix is buried within the core and protected. Another example of substantial and unexpected exchange-rate variation is within the less conserved but essential nucleobase binding loop (Fig. 6), which encompasses essential residues Tyr^{288} and Ser^{292} (fragment 20). The former stacks against the adenine base, whereas the latter donates a hydrogen bond to the N7 position of the purine base, making the ATPase purine-specific (30). In the apoprotein, neither of the two residues is engaged in these interactions. Although both residues are part of the same β-hairpin, Tyr^{288} is as unprotected as the adjacent loop, whereas Ser^{292} exchanges with an intermediate rate. Based on comparison with nucleotide-bound states of φ8 P4 (21), it is expected that these exchange rates will be sensitive to the nucleotide binding.

Another important insight from the predictions is that exchange at short times provides additional, valuable information about the dynamics of the system that cannot be inferred from exchange at longer times. Most common HDX-MS experiments, such as those available for φ12 P4 studied here, rely on manual mixing, and the shortest time at which the fraction of deuterium exchanged is measured is of the order of ≥ 10 s. The estimation of the kinetics of deuterium exchange on subsecond times (Fig. 3 *A*) reveals that fragments with similar exchange kinetics on the timescale of, e.g., seconds and minutes, may have very different kinetics at shorter times. A pertinent case is the comparison between the kinetics of experimentally observed fragment 20 (residues 284–293) and the fragment consisting of residues 292–302. Their kinetics are almost identical in the range 30 s to 4 h, whereas they are clearly distinguishable on a longer timescale. In fact, a time resolution of ~10–100 ms, accessible by a conventional rapid-quench-flow apparatus (38), would cover the relevant exchange kinetics, whereas little information would be obtained on a shorter timescale. This timescale is also relevant to the overall turnover rate (~6 s^{-1}) of the enzyme and quantitative prediction of exchange from a population of modeled states on this time scale will be essential in making use of HDX to monitor and interpret conformational changes associated with motor action.

Most of the theoretical models interpret HDX exchange kinetics obtained by NMR at the residue level for relatively small proteins (11). Only a few methods have attempted to predict deuteration measured by HDX-MS and these were limited to comparison with experimental data at only one time point (26,38). As illustrated here, reliable simulations of the entire experimental kinetics allow extraction of the residue-specific protection factors at different amide sites within each fragment (see, e.g., Fig. 6), thus enriching information content of the HDX-MS results and providing a direct link to the sequence, e.g., by informing site-directed mutagenesis experiments.

The COREX (25) method is based on populating protein microstates in which each residue is in either a fully folded (protected) or fully unfolded (exchangeable) state. The contribution of these microstates to exchange is then weighted according to their relative stability. This method, albeit computationally intensive, is effective in predicting HDX-MS. One limitation is that in its present form, the COREX approach ignores regions that are not resolved in the high-resolution structure. In addition to missing predictions for such regions, this omission from the model may affect exchange of the neighboring sites. In our approach, this issue is dealt with by modeling the missing regions within the context of the whole structure, using MD to relax the model, and, it is important to note, calculating protection factors as Boltzmann averages. However, as illustrated by the C-terminal helix case here, the quality of the initial model plays a crucial role in the success of this approach, since the relatively short duration of the MD run does not account for larger conformational changes that occur on longer timescales. An iterative approach in which different models of the missing regions are tested and the simulation results compared with experiment may yield a complete, plausible structure.

SUPPORTING MATERIAL

Supporting Methods, six figures, and two tables are available at http://www.biophysj.org/biophysj/supplemental/S0006-3495(14)00685-7.

This work was supported by a Wellcome Trust PhD studentship (096686/Z/11/Z) to G.R.

REFERENCES

1. Karplus, M., and J. A. McCammon. 2002. Molecular dynamics simulations of biomolecules. *Nat. Struct. Biol.* 9:646–652.
2. Karplus, M., and J. Kuriyan. 2005. Molecular dynamics and protein function. *Proc. Natl. Acad. Sci. USA.* 102:6679–6685.
3. Frauenfelder, H., G. Chen, ..., R. D. Young. 2009. A unified model of protein dynamics. *Proc. Natl. Acad. Sci. USA.* 106:5129–5134.
4. Huang, Y. J., and G. T. Montelione. 2005. Structural biology: proteins flex to function. *Nature.* 438:36–37.
5. Englander, J. J., C. Del Mar, ..., V. L. Woods, Jr. 2003. Protein structure change studied by hydrogen-deuterium exchange, functional labeling, and mass spectrometry. *Proc. Natl. Acad. Sci. USA.* 100:7057–7062.
6. Konermann, L., J. Pan, and Y. H. Liu. 2011. Hydrogen exchange mass spectrometry for studying protein structure and dynamics. *Chem. Soc. Rev.* 40:1224–1234.
7. Clarke, J., and A. R. Fersht. 1996. An evaluation of the use of hydrogen exchange at equilibrium to probe intermediates on the protein folding pathway. *Fold. Des.* 1:243–254.
8. Krishna, M. M., L. Hoang, ..., S. W. Englander. 2004. Hydrogen exchange methods to study protein folding. *Methods.* 34:51–64.
9. Morozova, L. A., D. T. Haynie, ..., C. M. Dobson. 1995. Structural basis of the stability of a lysozyme molten globule. *Nat. Struct. Biol.* 2:871–875.
10. Vendruscolo, M., E. Paci, ..., M. Karplus. 2003. Rare fluctuations of native proteins sampled by equilibrium hydrogen exchange. *J. Am. Chem. Soc.* 125:15686–15687.

11. Dempsey, C. E. 2001. Hydrogen exchange in peptides and proteins using NMR spectroscopy. *Prog. Nucl. Magn. Reson. Spectrosc.* 39:135–170.

12. Zhang, Z., and D. L. Smith. 1993. Determination of amide hydrogen exchange by mass spectrometry: a new tool for protein structure elucidation. *Protein Sci.* 2:522–531.

13. Katta, V., and B. T. Chait. 1993. Hydrogen/deuterium exchange electrospray ionization mass spectrometry: a method for probing protein conformational changes in solution. *J. Am. Chem. Soc.* 115:6317–6321.

14. Lam, T. T., J. K. Lanman, …, P. E. Prevelige. 2002. Mapping of protein:protein contact surfaces by hydrogen/deuterium exchange, followed by on-line high-performance liquid chromatography-electrospray ionization Fourier-transform ion-cyclotron-resonance mass analysis. *J. Chromatogr. A.* 982:85–95.

15. Kan, Z. Y., L. Mayne, …, S. W. Englander. 2011. ExMS: data analysis for HX-MS experiments. *J. Am. Soc. Mass Spectrom.* 22:1906–1915.

16. Suchanova, B., and R. Tuma. 2008. Folding and assembly of large macromolecular complexes monitored by hydrogen-deuterium exchange and mass spectrometry. *Microb. Cell Fact.* 7:12.

17. Landgraf, R. R., M. J. Chalmers, and P. R. Griffin. 2012. Automated hydrogen/deuterium exchange electron transfer dissociation high resolution mass spectrometry measured at single-amide resolution. *J. Am. Soc. Mass Spectrom.* 23:301–309.

18. Kan, Z. Y., B. T. Walters, …, S. W. Englander. 2013. Protein hydrogen exchange at residue resolution by proteolytic fragmentation mass spectrometry analysis. *Proc. Natl. Acad. Sci. USA.* 110:16438–16443.

19. Huang, R. Y., K. Garai, …, M. L. Gross. 2011. Hydrogen/deuterium exchange and electron-transfer dissociation mass spectrometry determine the interface and dynamics of apolipoprotein E oligomerization. *Biochemistry.* 50:9273–9282.

20. Lísal, J., D. E. Kainov, …, R. Tuma. 2006. Interaction of packaging motor with the polymerase complex of dsRNA bacteriophage. *Virology.* 351:73–79.

21. Lísal, J., T. T. Lam, …, R. Tuma. 2005. Functional visualization of viral molecular motor by hydrogen-deuterium exchange reveals transient states. *Nat. Struct. Mol. Biol.* 12:460–466.

22. Skinner, J. J., W. K. Lim, …, S. W. Englander. 2012. Protein dynamics viewed by hydrogen exchange. *Protein Sci.* 21:996–1005.

23. Lobanov, M. Y., M. Y. Suvorina, …, O. V. Galzitskaya. 2013. A novel web server predicts amino acid residue protection against hydrogen-deuterium exchange. *Bioinformatics.* 29:1375–1381.

24. Hilser, V. J., and E. Freire. 1996. Structure-based calculation of the equilibrium folding pathway of proteins. Correlation with hydrogen exchange protection factors. *J. Mol. Biol.* 262:756–772.

25. Liu, T., D. Pantazatos, …, V. L. Woods, Jr. 2012. Quantitative assessment of protein structural models by comparison of H/D exchange MS data with exchange behavior accurately predicted by DXCOREX. *J. Am. Soc. Mass Spectrom.* 23:43–56.

26. Petruk, A. A., L. A. Defelipe, …, A. G. Turjanski. 2013. Molecular dynamics simulations provide atomistic insight into hydrogen exchange mass spectrometry experiments. *J. Chem. Theory Comput.* 9:658–669.

27. Paatero, A. O., L. Mindich, and D. H. Bamford. 1998. Mutational analysis of the role of nucleoside triphosphatase P4 in the assembly of the RNA polymerase complex of bacteriophage ϕ6. *J. Virol.* 72:10058–10065.

28. Kainov, D. E., E. J. Mancini, …, R. Tuma. 2008. Structural basis of mechanochemical coupling in a hexameric molecular motor. *J. Biol. Chem.* 283:3607–3617.

29. Mancini, E. J., D. E. Kainov, …, D. I. Stuart. 2004. Atomic snapshots of an RNA packaging motor reveal conformational changes linking ATP hydrolysis to RNA translocation. *Cell.* 118:743–755.

30. El Omari, K., C. Meier, …, E. J. Mancini. 2013. Tracking in atomic detail the functional specializations in viral RecA helicases that occur during evolution. *Nucleic Acids Res.* 41:9396–9410.

31. Mancini, E. J., and R. Tuma. 2012. Mechanism of RNA packaging motor. *Adv. Exp. Med. Biol.* 726:609–629.

32. Kainov, D. E., R. Tuma, and E. J. Mancini. 2006. Hexameric molecular motors: P4 packaging ATPase unravels the mechanism. *Cell. Mol. Life Sci.* 63:1095–1105.

33. Bai, Y., J. S. Milne, …, S. W. Englander. 1993. Primary structure effects on peptide group hydrogen exchange. *Proteins.* 17:75–86.

34. Englander, S. W., T. R. Sosnick, …, L. Mayne. 1996. Mechanisms and uses of hydrogen exchange. *Curr. Opin. Struct. Biol.* 6:18–23.

35. Milne, J. S., L. Mayne, …, S. W. Englander. 1998. Determinants of protein hydrogen exchange studied in equine cytochrome *c*. *Protein Sci.* 7:739–745.

36. Craig, P. O., J. Lätzer, …, P. G. Wolynes. 2011. Prediction of native-state hydrogen exchange from perfectly funneled energy landscapes. *J. Am. Chem. Soc.* 133:17463–17472.

37. Bahar, I., A. Wallqvist, …, R. L. Jernigan. 1998. Correlation between native-state hydrogen exchange and cooperative residue fluctuations from a simple model. *Biochemistry.* 37:1067–1075.

38. Truhlar, S. M., C. H. Croy, …, E. A. Komives. 2006. Solvent accessibility of protein surfaces by amide H/2H exchange MALDI-TOF mass spectrometry. *J. Am. Soc. Mass Spectrom.* 17:1490–1497.

39. Best, R. B., and M. Vendruscolo. 2006. Structural interpretation of hydrogen exchange protection factors in proteins: characterization of the native state fluctuations of CI2. *Structure.* 14:97–106.

40. Fersht, A. 1985. Enzyme Structure and Mechanism. W. H. Freeman, New York.

41. Austin, R. H., K. W. Beeson, …, I. C. Gunsalus. 1975. Dynamics of ligand binding to myoglobin. *Biochemistry.* 14:5355–5373.

42. Bai, Y., T. R. Sosnick, …, S. W. Englander. 1995. Protein folding intermediates: native-state hydrogen exchange. *Science.* 269:192–197.

43. Sali, A., and T. L. Blundell. 1993. Comparative protein modelling by satisfaction of spatial restraints. *J. Mol. Biol.* 234:779–815.

44. Brooks, B. R., C. L. Brooks, 3rd, …, M. Karplus. 2009. CHARMM: the biomolecular simulation program. *J. Comput. Chem.* 30:1545–1614.

45. Huiskonen, J. T., H. T. Jäälinoja, …, S. J. Butcher. 2007. Structure of a hexameric RNA packaging motor in a viral polymerase complex. *J. Struct. Biol.* 158:156–164.

Supplementary information for "Functional dynamics of hexameric helicase probed by hydrogen exchange and simulation" by Radou, Dreyer, Tuma and Paci.

Verification of the assignment of the m/z peaks

To verify whether mismatches between experimental and predicted exchange kinetics of some fragments were due to misassignment, we double-checked the assignment of the raw MS peaks. The mass of all possible fragments of the primary sequence between length 5 and 40 aminoacids was calculated by summing the residue masses and adding the mass of a water molecule (18.01056 u), corresponding to the adding of OH and H during the hydrolysis of the new C- and N-terminus, respectively. Each mass experimentally detected was compared with the calculated ones. A threshold precision of 0.05u was used, i.e. the theoretical peak was considered to potentially match with the experimental one if $|m_{exp}-m_{th}|=\Delta m$ was lower than 0.05u (see Table S2).

Identification of the fragments located at the interface:

The variation of solvent accessible surface area from the monomeric to the hexameric form for a fragment j, ΔS_j, was quantified as:

$$\Delta S_j = \frac{1}{n_j} \sum_{i=m_j}^{m_j+n_j-1} \frac{S_i^{mono} - S_i^{hexa}}{S_i^{mono}} \qquad \text{S1}$$

where S_i^{mono} and S_i^{hexa} are the total solvent accessible surface area of the residue i in the monomeric and hexameric structure, respectively. Solvent accessible solvent accessible surface was calculated using NACCESS with a probe radius of 1.4 Å.

Quantification of the matching between the predicted and measured fragments kinetics

The overall deviation between the experimental and the simulated deuteration kinetics was estimated through the mean square deviation defined as

$$\chi^2 = \frac{1}{N} \sum_{j=1}^{N} \frac{1}{T_j} \sum_{t=1}^{T_j} (D_j^{exp}(t) - D_j^{sim}(t))^2 \qquad \text{S2}$$

where N is the total number of fragments, T_j the total number of experimental time points for the fragment j, D_j^{exp} the experimental deuterium fraction of the fragment j at time t and D_j^{sim} the simulated deuterium fraction of the fragment j at time t.

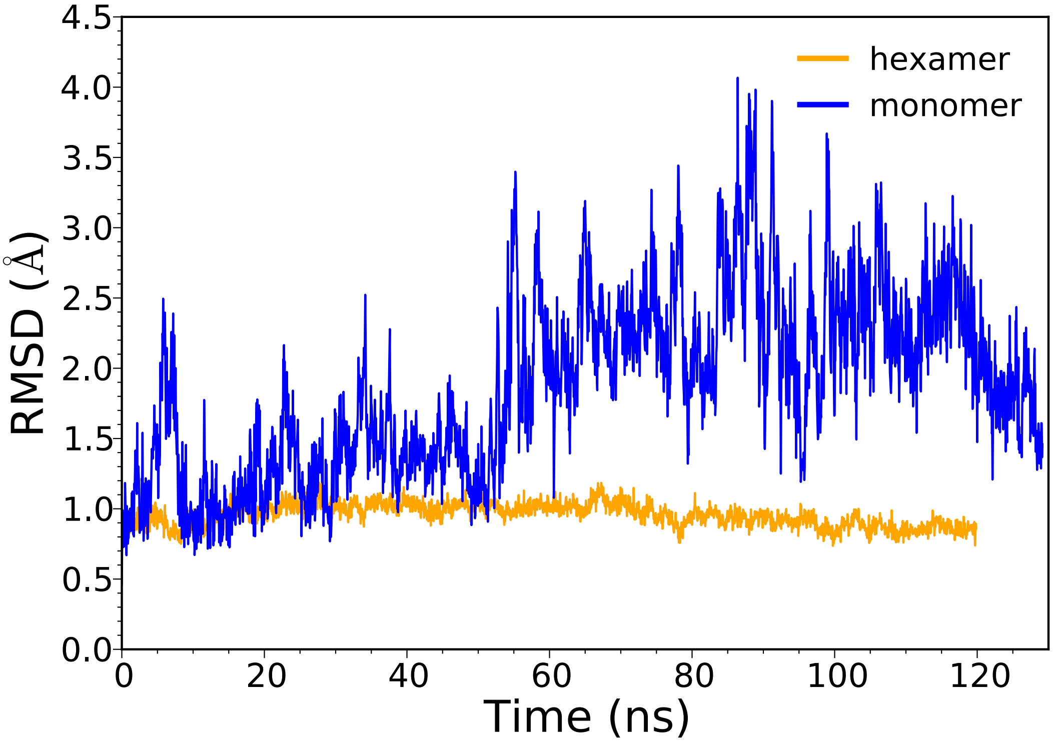

Figure S1. Root mean square deviation from the X-crystal structure (RMSD) of the monomer (blue) and the hexamer (orange), along the molecular dynamics simulation. The RMSD of the monomer stabilises around 2.5 Å, a considerably larger value than that observed for the average monomer in the hexamer (1 Å). The simulation suggests that the monomer native state is stable in solution but slightly deformed (especially at the N-terminus) and more fluctuating relative to the monomer in the hexamer. The simulation of the monomer was performed as described for the hexamer, at the same pressure and temperature, but in a smaller water box containing 24970 TIP3P molecules.

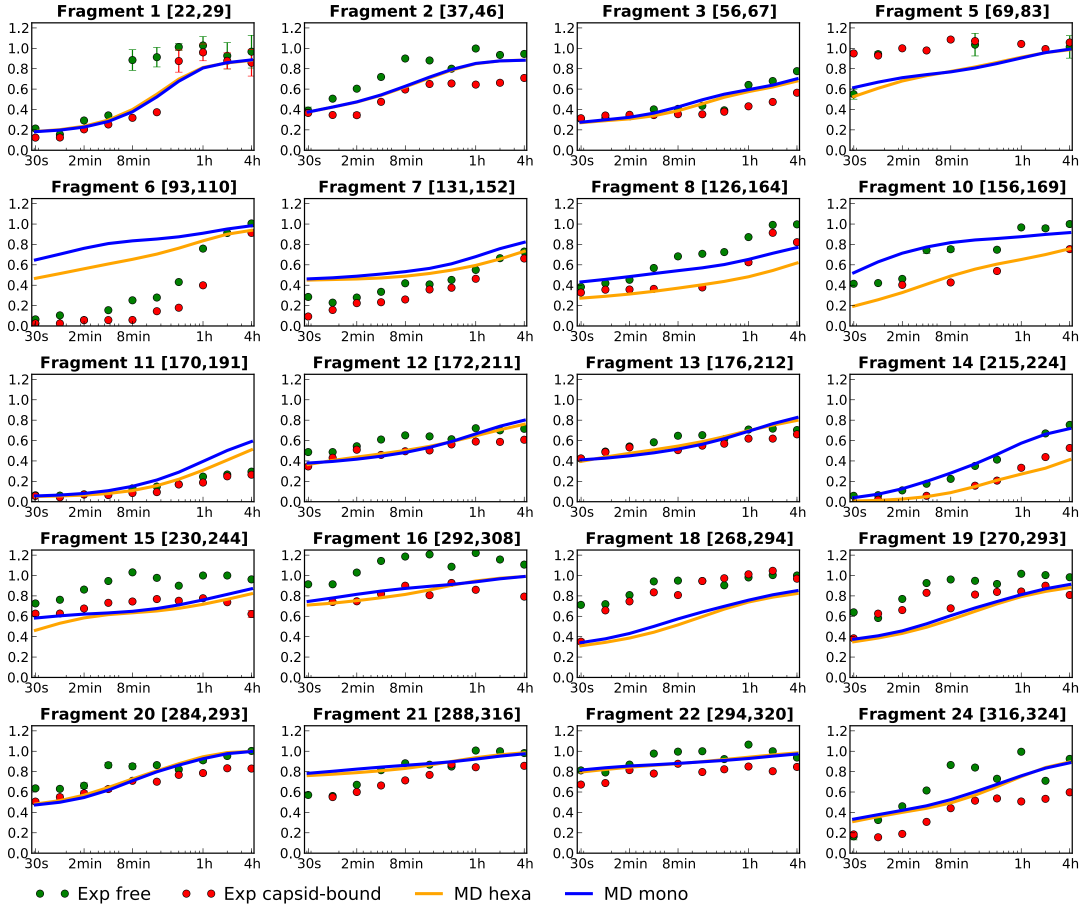

Figure S2. Predicted deuterium fractions (averages over the MD simulation) for the monomer (blue line) and the hexamer (orange line). The experimental deuterium fractions of the free hexamer and the hexamer assembled with the procapsid are shown as green and red dots, respectively. Experimental error bars are shown when larger than the symbols.

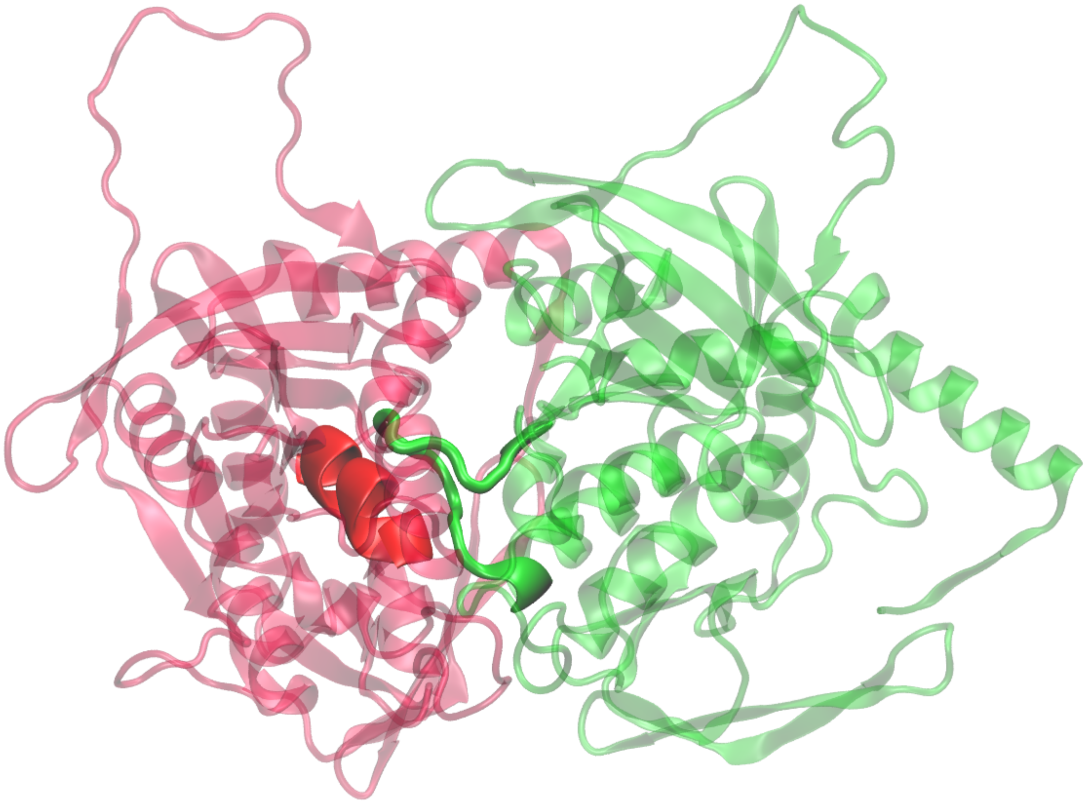

Figure S3. Cartoon representation of the interface between two neighbouring subunits of the hexamer. The fragments 14 and 10 are highlighted in red and green, respectively. For better clarity, the subunits are depicted up-side-down compare to Figures 1 and 4.

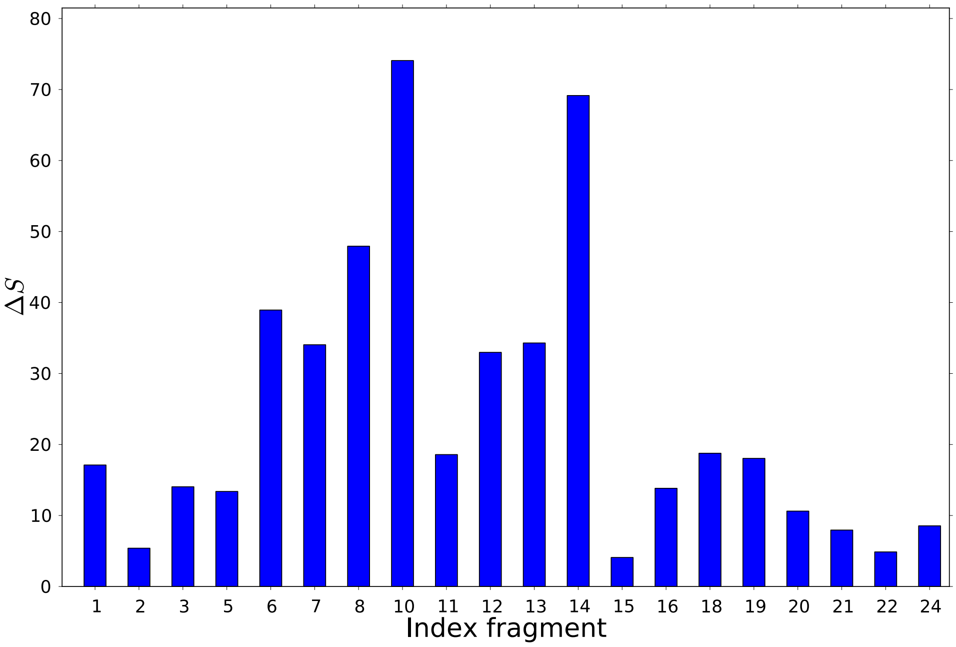

Figure S4. Change of the solvent accessible surface area of each fragment between the monomer and hexamer in the crystal structure.

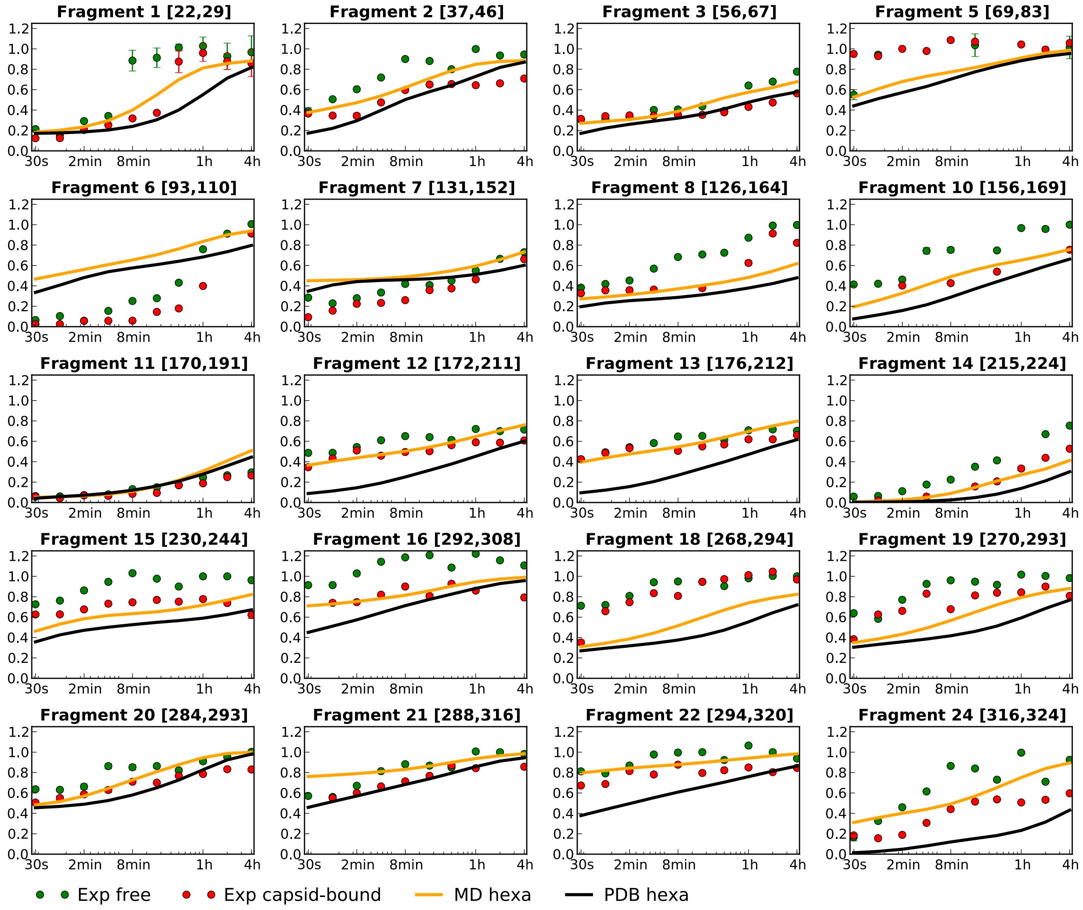

Figure S5. Exchange kinetics of the hexamer with (orange line) or without (black line) dynamics predicted from the MD simulations. The experimental fraction of the free hexamer and the hexamer assembled with the procapsid are represented as green and red dots, respectively. Experimental error bars are shown when larger than the symbols.

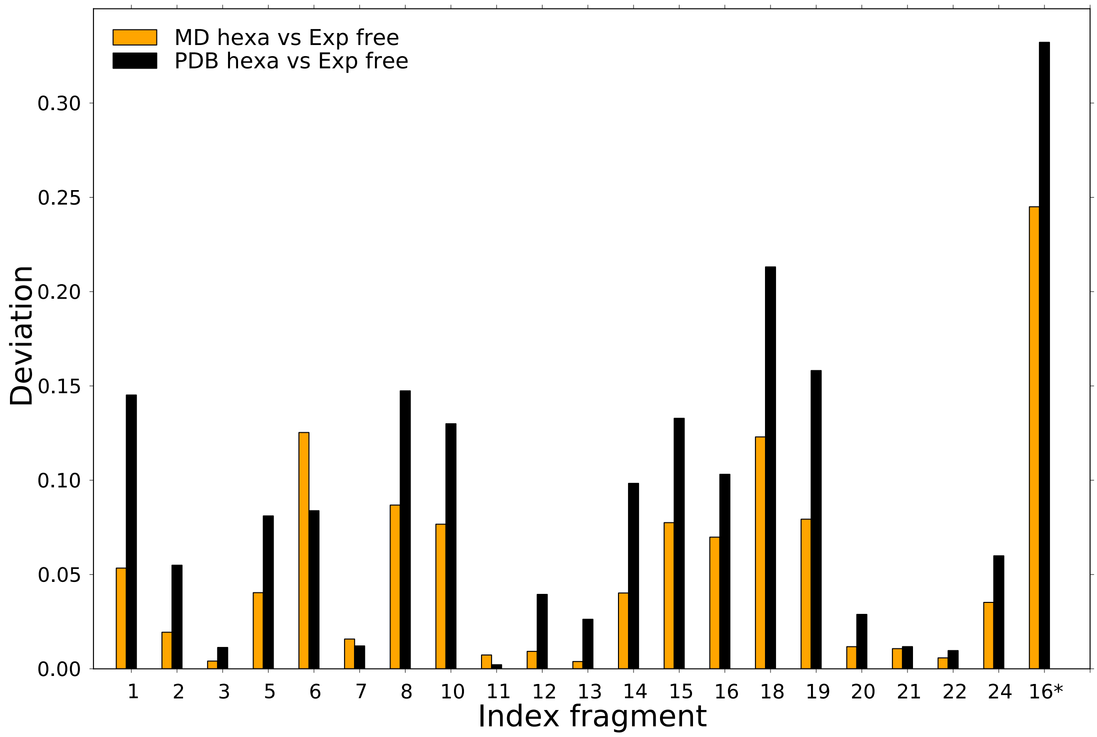

Figure S6. Deviation (χ^2 defined in Eq S2) of the HDX kinetics from the experimental data for the free hexamer as defined by Equation S2, for each fragment. Deviation has been calculated for the crystal structure (black) or the MD simulation of the hexamer (orange). Deviation for the old assignment of the fragment 16 is indicated with *.

List of fragments

Fragment number	Assignment
1	H33-W29
2	L37-V46
3	A56-V67
4	A56-V70
5	Y69-V83
6	Q93-S110
7	K131-K152
8	V126-G164
9	V126-G164
10	V156-F169
11	N170-L191
12	F172-A211
13	I176-F212
14	L215-S224
15	I230-E244
16	S292-S308
16*	I230-L245
17	D239-V246
18	Q268-L294
19	L270-V293
20	L284-V293
21	Y288-I316
22	L294-E320
23	E290-S330
24	I316-V324

Table S1. List of the fragments experimentally probed. The assignment of the fragment 16 proposed here and that reported in Ref [1] are called “new” and “old”, respectively.

	Experimental data			Old assignment	New assignment		
Id	m/z	z	exp-mass	sequence	sequence	mass	Δm
1	870.3828	1	869.37498	22-29	22-29	869.36676	0.00822
2	1045.5247	1	1044.51688	37-46	36-45	1044.49749	0.01939
					37-46	1044.49749	0.01939
					38-47	1044.49749	0.01939
					98-106	1044.54913	0.03225
					128-138	1044.55635	0.03947
3	687.4032	2	1372.79076	56-67	56-67	1372.78265	0.00811
					57-68	1372.78265	0.00811
4	868.5086	2	1735.00156	56-70	56-70	1734.97805	0.02351
					173-187	1734.95627	0.04529
5	813.4265	2	1624.83736	69-83	69-83	1624.83078	0.00658
					168-181	1624.79189	0.04547
6	963.9651	2	1925.91456	93-110	93-110	1925.94174	0.02718
7	726.413	3	2176.21554	131-152	131-152	2176.19635	0.01919
8	785.6373	5	3923.1474	126-164	126-164	3923.12678	0.02062
9	981.7956	4	3923.15112	126-164	126-164	3923.12678	0.02434
10	801.3862	2	1600.75676	156-169	156-169	1600.75214	0.00462
					160-173	1600.74091	0.01585
					38-53	1600.78315	0.02639
11	842.1326	3	2523.37434	170-191	170-191	2523.36311	0.01123
12	1020.807	4	4079.19672	172-211	172-211	4079.16974	0.02698
					173-212	4079.16974	0.02698
13	938.525	4	3750.06872	176-212	176-212	3750.04745	0.02127
14	935.4548	1	934.44698	215-224	215-224	934.44296	0.00402
					117-125	934.40792	0.03906
					206-214	934.4872	0.04022
					290-297	934.48721	0.04023
15	808.4156	2	1614.81556	230-244	230-244	1614.81005	0.00551
					231-245	1614.81005	0.00551
					238-251	1614.85766	0.0421
16	864.9627	2	1727.90976	230-245	292-308	1727.91659	0.00683
					140-156	1727.92058	0.01082
					230-245	1727.89411	0.01565
17	930.5123	1	929.50448	239-246	239-246	929.50693	0.00245
					312-319	929.51818	0.0137
					81-89	929.48179	0.02269
					142-151	929.48177	0.02271
					305-313	929.52941	0.02493
18	756.905	4	3023.58872	268-294	268-294	3023.57881	0.00991
					257-283	3023.56756	0.02116
					258-284	3023.56756	0.02116
19	671.8521	4	2683.37712	270-293	269-292	2683.36776	0.00936
					270-293	2683.36776	0.00936
					271-294	2683.36776	0.00936
					272-295	2683.36776	0.00936
					42-67	2683.41802	0.0409
20	1120.5242	1	1119.51638	284-293	284-293	1119.51964	0.00326
					285-294	1119.51964	0.00326
					258-267	1119.50839	0.00799
					17-26	1119.4985	0.01788
					55-64	1119.55601	0.03963
					280-289	1119.55603	0.03965
					106-116	1119.5634	0.04702
21	1009.2095	3	3024.60504	288-316	288-316	3024.5992	0.00584
21					292-320	3024.62033	0.01529
22	710.6451	4	2838.54912	294-320	294-320	2838.51989	0.02923
22					295-321	2838.51989	0.02923
23	1112.3603	4	4445.40992	290-330	290-330	4445.39897	0.01095
24	1046.5303	1	1045.52248	316-324	315-323	1045.52913	0.00665
					316-324	1045.52913	0.00665
					317-325	1045.52913	0.00665
					263-271	1045.508	0.01448

Table S2. Assignment for each fragment. In each column is indicated the index of the fragment, the experimental monoisotopic mass of the fragment, the number of charge, the previous assignment, the first residue of the new assignment, the last residue of the new assignment, the

monoisotopic mass of the new fragment, the absolute difference of mass between the experimental and predicted mass of the new fragment, respectively.

1. Lísal, J., et al., *Interaction of packaging motor with the polymerase complex of dsRNA bacteriophage.* Virology, 2006. **351**(1): p. 73-79.
